# The Finkelstein–Schoenfeld Test: A Note on Some Overlooked Issues Concerning Power

**DOI:** 10.1007/s43441-023-00608-z

**Published:** 2024-02-05

**Authors:** Rong Tang, Wei-Chen Chen, Heng Li, Nelson Lu, Yu Zhao

**Affiliations:** Center for Devices and Radiological Health, Food and Drug Administration, 10903 New Hampshire Avenue, Silver Spring, MD 20993 USA

**Keywords:** Finkelstein–Schoenfeld, Composite endpoint, Clinical trial, Time-to-event, Correlation, Study power

## Abstract

In this note, we express our viewpoint regarding power considerations, via simulation studies, in clinical study design using hierarchical composite endpoint and Finkelstein–Schoenfeld test.

## Introduction

The primary effectiveness endpoint is fundamental to the design of a randomized controlled trial (RCT) for a new medical intervention. It characterizes the clinical impact of the intervention and serves as a basis for medical decision-making. Traditionally, mortality is a commonly used primary effectiveness endpoint for a severe medical condition. In recent years, however, we have seen more widespread usage of composite endpoints as primary, defined in terms of time to the first event of a combination of death and clinically significant but non-fatal events such as stroke and myocardial infarction. Each individual event is called a component. One commonly cited advantage of such a composite endpoint is its potential to increase trial efficiency: the addition of the non-fatal events elevates the incidence rate and may boost the absolute size of treatment effect if the treatment has a positive effect on all components, in which case the requisite sample size would be reduced. However, as statistical testing for such a composite endpoint is based on time to first event, it gives events with different clinical significance equal priority. Consequently, statistical significance may be entirely attributable to components of lesser importance. Moreover, as non-fatal event occurs before death, the absence of effect on non-fatal events may make it difficult to see any survival benefit of the treatment.

Finkelstein and Schoenfeld introduced the idea of prioritized composite endpoint 20 years ago [1]. They proposed a Finkelstein–Schoenfeld test (FS test for short) in which the components of a composite endpoint are arranged into a hierarchy with the clinically most important event (usually death) on the top of the hierarchy and thus being given the highest priority. The second most important event is placed at the second level of the hierarchy, and so on and so forth. The FS test is carried out through pairwise comparisons between each pair of patients according to the pre-specified hierarchy regarding each component. This test also has the capability of combining different types of components, for example time-to-event variable, ordinal variable and continuous variable, and different components do not need to be measured contemporaneously.

The FS test is becoming more popular owing to its flexibility, built-in prioritization, as well as the potential to reduce sample size when the mortality benefit of the new intervention is modest but is accompanied by benefits on other components (non-fatal events and quality-of-life measures) [2]. Despite its increased uptake, the FS test has not been extensively studied for its statistical properties related to the sample size and power analysis. In current practice, sample size calculation is conducted via simulation. Depending on the types and number of components, multiple parameters are assumed in the simulation, such as treatment-specific means and variances (or event rates) for each component, and correlations between these components. On these correlations one may only have very scant information, and not much is known regarding the impact of correlation on the study efficiency. To bring more clarity to this issue, the intricate interplay between design parameters and study power is explored in this article via simulation studies. Specifically, this research is devoted to exploring whether the correlation between components can affect the efficiency of the study and whether a FS endpoint would be more efficient if it had more components with positive treatment effect. In the Materials and Methods section, a brief review of the FS test is provided, followed by description of a clinical trial scenario with a FS primary endpoint and the setting of the simulation studies. Simulation results are presented in Results section. In the Conclusion section, the findings are summarized, and some of the advantages and challenges of FS method and our recommendation are provided.

## Materials and Methods

### A Brief Introduction to the Finkelstein–Schoenfeld Method

In the FS test, the relative clinical importance of the components is built into the computation of the test statistic, which is based on pairwise comparison of all pairs of subjects. Below is a description of how the test statistic is computed.

Consider a randomized controlled trial with n subjects in the test arm and m subjects in the control arm. Define a comparison score ${u}_{ij}$ for each pair of subjects $i$ and $j$ in the trial as follows. Subjects $i$ and $j$ are compared on the component at the top of the hierarchy (usually mortality) first, and ${u}_{ij}$ is equal to + 1 if subject $i$ has lived longer and − 1 if subject $j$ has lived longer. If it cannot be determined which subject has lived longer (such as when both subjects are censored or missing), then they are compared on the component at the second level of the hierarchy using similar strategy. The comparison continues down the hierarchy until a patient with a better outcome can be decided (with ${u}_{ij}$ equal to + 1 if subject $i$ has better outcome and − 1 if subject $j$ has better outcome). If the last component is reached and a decision still cannot be made, then ${u}_{ij}$ is equal to 0. It should be noted that ${u}_{ji}={-u}_{ij}$ for all $i\ne j$. After each pair of subjects $i$ and $j$ is assigned a value ${u}_{ij}$ of either + 1, − 1 or 0, a score ${U}_{i}$ is computed for each subject $i$ in the trial, which is equal to the sum of ${u}_{ij}$ over all $j\ne i$. Finally, the test statistic $T$ is equal to the sum of ${U}_{i}$ over subjects in the test arm. In essence, the FS test is a randomization test based on permutation of treatment status with the null hypothesis being that patients have the same outcome regardless of the treatment received.

### A Hypothetical Clinical Scenario

The FS endpoint is often used in heart failure studies where the target patient population is at high risk of one or more of (1) debilitating clinical symptoms, (2) hospitalization or (3) death. It is commonly believed that a useful treatment for heart failure should provide benefit in at least one of the three areas. In the following example, a randomized controlled study is designed to study the effectiveness of a new treatment in heart failure patients. To fully evaluate the clinical course of the patients and possibly have a more efficient study, a FS endpoint is utilized to incorporate the following components hierarchically into a single metric:

- Time to all cause death
- Time to heart failure hospitalization
- Change in Kansas City Cardiomyopathy Questionnaire (KCCQ) [3] from the baseline to 12 months.

Some assumptions are needed when estimating the sample size. The all-cause mortality rate for the treatment and control groups at 1 year are assumed to be 13% and 15%, respectively. The annual HF hospitalization rate is assumed to be 15% for the treatment group and 25% for the control group. Meanwhile, the mean change in 12-month KCCQ from the baseline in treatment group is assumed to be 7.5 and 0 in the control group, standard deviation is set to 20 for both treatment groups. As the analytical form of FS endpoint sample size calculation is not available, simulation is needed to determine the appropriate sample size to adequately power the study. Based on the abovementioned assumptions and 1:1 randomization, a sample size (*N*) of 400 would provide 85% power under the assumption that the components are independent.

However, ignoring the correlation among the components is questionable as it may lead one to obtain an ill-advised sample size when the components are in fact correlated. Some studies suggest that hospitalization for heart failure (HF) may be associated with increased risk of death among patients with heart failure [4, 5]. If correlations exist among some or all components of a FS endpoint, an inaccurate power may be obtained when ignoring such a correlation structure.

Another consideration, from a pure statistical perspective, is whether it is always beneficial to increase the number of components with positive treatment effect. In this example, the first component, mortality, holds the highest clinical relevance, but the treatment effect between the two arms is relatively small. If the endpoint only includes this component, a large sample size may be required to obtain a certain power. However, when incorporating other components into the endpoint, it is often believed that the power would be increased with every additional component. The question is whether it is always true that a FS endpoint is more efficient if it has more components with positive treatment effect.

### Settings for Simulation Studies

To explore and gain better understanding of the potential impact of additional component(s) as well as between-component correlations of FS endpoint on Type I error rate and study power, simulation studies have been conducted based on the FS endpoints described in the previous section.

Suppose that a two-arm 1:1 randomized controlled trial (RCT) including 400 HF patients is proposed to demonstrate the superiority of a subject device comparing to a control device regarding the FS endpoint. The first component, time to all-cause death, is assumed to follow an exponential distribution. The 1-year death rates are assumed to be ${d}_{t}$ and ${d}_{\text{c}}$ for the treatment and the control groups, respectively. Similarly, the second component, time to HF hospitalization, is assumed to be exponentially distributed with 1-year HF hospitalization rate of ${h}_{t}$ in the treatment group and ${h}_{\text{c}}$ in the control group. The third component, 1-year change in KCCQ from baseline, is denoted by $K$ and assumed to be normally distributed with means of ${mk}_{t}$ and ${mk}_{\text{c}}$ in the treatment and control groups, respectively, with the common standard deviations of 20. Simulations were conducted under several scenarios with different values ${d}_{t}$, ${d}_{\text{c}}$, ${h}_{t}$, ${h}_{\text{c}}$, ${mk}_{t}$, and ${mk}_{\text{c}}$.

To simulate data with between-component correlation, the following approach was used. For each subject, we first drew three components from a multivariate normal distribution with a variance–covariance matrix. Then through specific data transformation, each component was marginally converted to either a time-to-event or a continuous endpoint. After the transformation, the correlation levels between FS components do not change greatly from those set in the variance–covariance matrix of the multivariate normal distribution. Five correlation levels are specified in each scenario: independent, mild, moderate, moderately high, and high. The true censoring time is generated independently from the event time, following an exponential distribution with 1-year censoring rate of 20%. The details of simulation plan can be found in the Appendix.

A total of seven scenarios are considered in the simulation studies. In Scenario 1, it is assumed the there are no treatment effects in any components. For other scenarios, the treatment effect size for each component is assumed to be either mild or strong, as displayed in Table 1. The treatment effect size for component 1 (1-year death) is considered strong if ${d}_{t}/{d}_{\text{c}}\le 0.5$ and mild if ${0.5<d}_{t}/{d}_{\text{c}}<1$. The treatment effect for component 2 (time to HF hospitalization) is considered strong if ${h}_{t}/{h}_{\text{c}}\le 0.5$ and mild if $0.5<{h}_{t}/{h}_{\text{c}}<1$. The treatment effect for component 3 (1-year change in KCCQ from baseline) is considered strong if ${mk}_{t}-{mk}_{\text{c}}\ge 5$ and mild if $0<{mk}_{t}-{mk}_{\text{c}}<5$. A total of 2000 datasets were generated under each simulation scenario.

**Table 1.** Treatment Effect Sizes by Components per Simulation Scenario.

Component	Scenario						
	1	2	3	4	5	6	7
1-Year all-cause death	No	Strong	Mild	Strong	Mild	Mild	Mild
1-Year HF hospitalization rate	No	Strong	Strong	Mild	Mild	Mild	Strong
KCCQ	No	Mild	Strong	Mild	Strong	Mild	Mild

## Results and Discussion

### Impact of Correlation

To investigate the impact of the correlation among components to the type I error rate and power, simulations were conducted per Scenarios 1 to 7, and the results are displayed in Table 2. Based on Scenario 1 (under the null hypothesis), it can be observed that Type I error rate is preserved around 0.05 regardless of the magnitude of the between-component correlation. Similarly, when the subject device has only mild or moderate treatment effects in all three components, the study power is consistently low regardless of the levels of between-component correlations, as suggested by results of Scenario 6.

**Table 2.** Simulation Results for Impact of Correlations.

Scenario	${d}_{t}$	${d}_{\text{c}}$	${h}_{t}$	${h}_{\text{c}}$	${mk}_{t}$	${mk}_{\text{c}}$	Correlation level	Power
1	0.15	0.15	0.25	0.25	7.5	7.5	Independent	0.054
							Mild	0.047
							Moderate	0.051
							Moderately high	0.058
							High	0.052
2	0.06	0.15	0.10	0.25	7.5	5.0	Independent	0.991
							Mild	0.961
							Moderate	0.891
							Moderately high	0.743
							High	0.549
3	0.13	0.15	0.10	0.25	7.5	0	Independent	0.974
							Mild	0.940
							Moderate	0.910
							Moderately high	0.892
							High	0.892
4	0.06	0.15	0.22	0.25	7.5	5.0	Independent	0.650
							Mild	0.580
							Moderate	0.447
							Moderately high	0.342
							High	0.265
5	0.13	0.15	0.22	0.25	7.5	0.0	Independent	0.505
							Mild	0.494
							Moderate	0.534
							Moderately high	0.599
							High	0.695
6	0.13	0.15	0.22	0.25	7.5	5.0	Independent	0.191
							Mild	0.182
							Moderate	0.165
							Moderately high	0.172
							High	0.174
7	0.10	0.12	0.15	0.30	7.5	6.5	Independent	0.760
							Mild	0.640
							Moderate	0.530
							Moderately high	0.397
							High	0.299

Scenarios 2 and 3 are cases in which the subject device has substantially beneficial treatment effects on two of the FS components while only has subtle improvement on the other one. In both scenarios, the study power decreases as the level of between-component correlation rises. Particularly in Scenario 2, the power drastically shrinks from 97 to 50% when the between-component correlation increases. One possible explanation is that when the correlation between two components with strong treatment effects increases, the total information contained in these two components decreases, resulting in reduced study power.

Scenarios 4, 5, and 7 are cases in which the subject device only has noticeable beneficial treatment effect in one FS component. In Scenario 4, the power is only 65% when the components are independent, indicating the lack of power with given sample size of 400. As the level of between-component correlations increases, the study power goes down even further. From the results of Scenario 5, there is no clear trend of the study power by increasing the level of between-component correlations. The results of Scenario 7 show a similar decreasing pattern of power when the correlation level increases as observed in Scenario 4.

In summary, results from Table 2 suggest that between-component correlations could have dramatic impact on the study power. The direction and extent of this impact seems to be affected by the treatment effect on each component and the hieratical order of the FS components. Though a clear pattern has not been observed, these findings highlight the importance of considering various levels of the between-component correlations when estimating the sample size. In practice, clinical input is needed regarding the range of reasonable between-component correlation structures of a FS primary endpoint in the design stage.

### Benefits of Adding Components

To investigate the “benefit” of adding components to a FS endpoint to increase study power, simulations were conducted by sequentially adding the components based on Scenario 7.

Scenario 7a represents the case when the endpoint only includes the first component, Scenario 7b the first two components, and Scenario 7c all components. The results are displayed in Table 3. The components are assumed to be independent for simplicity. The power jumps greatly when the second component is added. However, it decreases a little after the third component is included.

**Table 3.** Simulation Results for Impact of Number of Components.

Scenario	${d}_{t}$	${d}_{\text{c}}$	${h}_{t}$	${h}_{\text{c}}$	${mk}_{t}$	${mk}_{\text{c}}$	Correlation level	Power
7a	0.10	0.12					Independent	0.104
7b	0.10	0.12	0.15	0.30			Independent	0.824
7c	0.10	0.12	0.15	0.30	7.5	6.5	Independent	0.760

Intuitively, adding a component with positive treatment effect to a FS endpoint would help increase the chance to demonstrate the superiority of the subject device. Some may view this simulation result somewhat surprising, as the test statistic of the FS method, $T$, likely increases with the added component. To see this, recall that the test statistic of the FS method is $T=\sum {U}_{i}$ where $i$ is the index for subjects in the subject device group and ${U}_{i}={\sum }_{j\ne i}{u}_{ij}$, $j$ is the index for all subjects. When a component with positive treatment effect is added to a FS endpoint as the lowest in the hierarchy, ${u}_{ij}$ originally with values of $\pm 1$ do not change, but ${u}_{ij}$ originally with values of 0 may remain the same or change to either 1 or − 1. It is more likely to turn into 1 due to the positive treatment effect of the new component. However, when $T$ increases, $V$, (the variance of $T$), may increase as well. As the p-value of FS method is calculated based on asymptotic normality of $T/\sqrt{V}$, the p-value (based on the FS endpoint with the additional component) may go either direction from the original p-value (without the additional component). The statistics based on a dataset from the simulation study is provided in Table 4 to illustrate this.

**Table 4.** The Statistics and p-Values of FS Tests Based on One Simulated Dataset for Components that are Sequentially Added to the FS Endpoint.

FS Endpoint	$T$	$\sqrt{V}$	Test statistics	p-value
Component 1 only	1021	975.20	1.047	0.1476
Components 1, 2	3055	1561.92	2.086	0.0252
Components 1, 2, 3	3130	1919.18	1.631	0.0515

In summary, this simulation study shows that adding a component does not necessarily increase the power. Sometimes, the power could even go down, even when there is some positive treatment effect regarding the added component.

### Power Analysis for the Hypothetical Scenario

**Figure 1.**
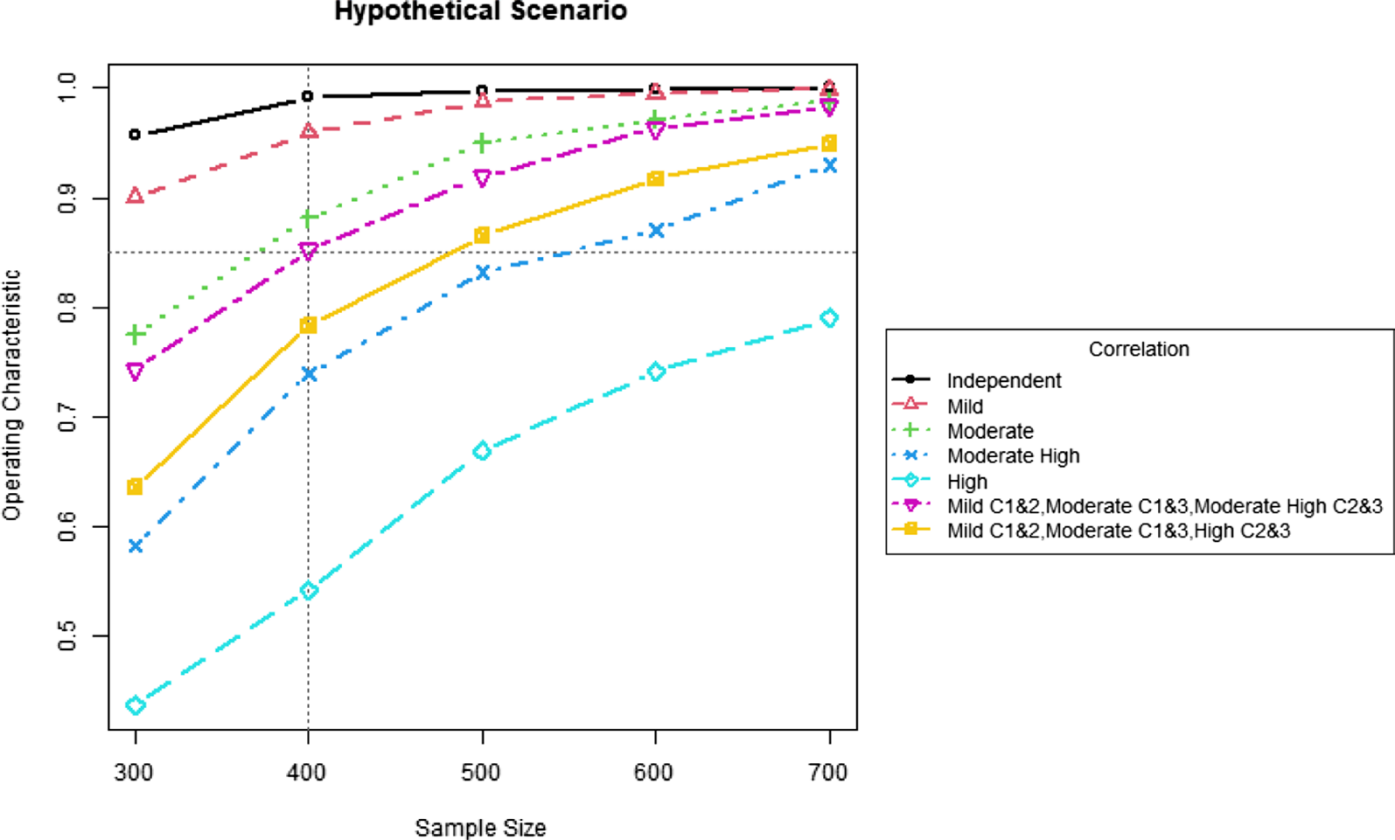
Power curves (powers vs. sample sizes) under different correlation levels for the hypothetical clinical scenario. “Independent”, “Mild”, “Moderate”, “Moderate High”, and “High” indicate the level of all between-component correlations. “Mild C1&2” indicates a mild correlation between components 1 and 2, “Moderate C1&3” indicates a moderate correlation between components 1 and 3. “Moderate High C2&3” and “High C2&3” indicates that correlations between components 2 and 3 are moderate high and high, respectively. For each power curve, the computing time of 2000 simulations roughly takes 0.33, 1.0, 2.7, 6.0, and 12.1 h for samples size 300, 400, 500, 600, and 700, respectively, using 16 parallel processors in a workstation of Linux environment (Intel Xeon CPU E5-2630 @2.30 GHz and 192 GB memory).

In this section, a power analysis is presented based on the hypothetical clinical scenario introduced earlier. Recall that the assumed parameters are ${d}_{t}=0.06$, ${d}_{\text{c}}=0.15$, ${h}_{t}=0.10$, ${h}_{\text{c}}=0.25$, ${mk}_{t}=7.5$ and ${mk}_{\text{c}}=5.0$, which is the setting of the Scenario 2 in the simulation study. In Table 2, the powers were obtained via simulation based on the fixed sample size of 400 under the assumed rates and different correlation levels. In this power analysis, powers are obtained for additional sample sizes (300, 500, 600, and 700) under the same settings, and the results of powers against sample sizes are displayed in Fig. 1. It can be observed that, if the correlation level is mild or non-existent, a sample size of 300 is adequate to obtain a power of 90% or more. If the correlation level is moderate, a power of 85% is attained with a sample size of close to 400. If the correlation level is moderately high, more than 500 subjects are required to obtain a power of 85%. If the correlation level is high, even 700 subjects are not adequate to obtain 85% power.

Additionally, we explored two situations where the correlation level between one pair of components is different from that between another pair. In one situation, the correlation between components 1 and 2 is mild, the correlation between components 1 and 3 is moderate, and the correlation between components 2 and 3 is moderately high. In the other situation, we assume mild correlation between components 1 and 2, moderate correlation between components 1 and 3 and high correlation between components 2 and 3. The resulting powers against various sample sizes are also displayed in Fig. 1. It can be observed that a power of 85% can be reached with a sample size of 500. Based on the assumed parameter values and correlation structure(s) among components, the study designer can decide the range of appropriate sample sizes to attain the desired power.

## Conclusion

The FS endpoint has gained popularity in clinical trials due to its capacity to differentiate components according to their clinical importance. However, the built-in hierarchical structure introduces additional complexity for power analysis, so the sample size calculation is commonly conducted through simulations in practice. Due to the multivariate nature of prioritized composite endpoints, the values of multiple parameters need to be specified in the simulation, including not only those governing the marginal distributions of individual component endpoints but also those governing the dependence structure between the component endpoints. Typically, the kind of prioritized composite endpoints discussed in this paper are constituted by both time-to-event endpoints and longitudinal endpoints. Estimates of the parameters governing the dependence structure between time-to-event endpoints, and between time-to-event endpoints and longitudinal endpoints, are seldom reported in the medical literature. So, what should one do with these parameters, which we refer to as “correlations,” in simulations for sample size planning? This question is particularly acute if it turns out that power is sensitive to the magnitude of these parameters. A main purpose of this paper is to explore with a small number of simulations the sensitivity of power to the correlations between component endpoints. In particular, the tables and graphs in this paper are not meant to serve as tools that the practitioner can use in their sample size calculation.

In this research, the first simulation study (Table 2) leads us to conclude that power may vary greatly with different magnitude of correlations between components. In addition, there is no consistent trend. That is, there is no guarantee that the higher the correlation, the smaller the power, or vice versa. The phenomenon of no clear trends may be attributed to factors such as the complexity of the built-in hierarchy, the magnitude of treatment effect for each component, and the underlying correlation structure between components. When conducting a simulation study to determine the sample size, our suggestion is to consider and explore a relatively wide range of the correlation structures to ensure that the study is adequately powered.

The second simulation study (Table 3) illustrates that incorporating additional components into a FS endpoint may not always boost the study power. Some researchers and trialists may intend to add components in the hope of increasing the chance to claim success, even though some of these components are much less clinical meaningful. However, this strategy may not only dilute the clinical meaningfulness of the FS endpoint, but also even hurt the study efficiency when there is only a minimal treatment effect for some of these components.

Our findings highlight the importance of close collaboration between statisticians and clinicians in the process of constructing a primary FS endpoint and necessity of comprehensive simulation studies at the design stage.

## Data Availability

This paper uses simulation to illustrate the impact of correlation, no real data is used.

## Appendix

To simulate data for the three FS components (${T}_{di}$, ${T}_{hi}$, ${K}_{i}$) and incorporate between-component correlations at the same time, the following data simulation approach was used. For each subject $i$, we first drew ${{\varvec{X}}}_{i}={({X}_{i1},{X}_{i2},{X}_{i3})}{\prime}$ from a multivariate normal distribution, $MVN({{\varvec{\mu}}}_{X},{{\varvec{\Sigma}}}_{X})$, with mean ${{\varvec{\mu}}}_{X}={(0, \mathrm{0,0})}{\prime}$ and variance–covariance matrix ${{\varvec{\Sigma}}}_{X}$. For simplicity, we also assumed the compound symmetric structure for the covariance matrix with the variance $1$ for all observations on the same subject $i$ and with the correlation, $\rho$, for all pairs of observations, ${X}_{ij}$ and ${X}_{ik}$ ($j\ne k$), on the same subject $i$.

Then through specific data transformation, ${X}_{i1}$, ${X}_{i2}$, and ${X}_{i3}$ were marginally converted to the uniform random variables via ${U}_{ij}\sim {\Phi }^{-1}({X}_{ij})$ for $j=1$ and $2$ where ${\Phi }^{-1}(\bullet )$ is the inverse cumulative distribution function of the standard normal distribution. Suppose the subject $i$ was randomized to the test arm, then the data of the subject $i$ were generated as following. First, the true time to all-cause death ${T}_{di}$ followed an exponential distribution, $Exp\left(-log(1-{d}_{t})\right)$, which was drawn from $(-log(1-{U}_{i1}))/(-log(1-{d}_{t}))$. Second, the true time to HF hospitalization ${T}_{hi}$ also followed an exponential distribution, $Exp\left(-log(1-{h}_{t})\right)$, which was drawn from $(-log(1-{U}_{i2}))/(-log(1-{h}_{t}))$. Third, the true KCCQ change ${K}_{i}$ followed a normal distribution, $N\left({mk}_{t},{20}^{2}\right)$, which was drawn from $20\times {X}_{i3}+{mk}_{t}$ (i.e., standard deviation sd = 20). Finally, the true censoring time ${C}_{i}$ followed an exponential distribution with 1-year censoring rate of 20% which was drawn from the exponential distribution function independently. Hence, the observed time to all-cause death and the observed time to HF hospitalization for each subject were set to be $min({T}_{di},{C}_{i})$ and $min({T}_{hi},{C}_{i})$, respectively. Similarly, the data for the control subjects were generated with the parameters ${d}_{\text{c}}$, ${h}_{\text{c}}$, and ${mk}_{\text{c}}$ by replacing the parameters ${d}_{t}$, ${h}_{t}$, and ${mk}_{t}$, aforementioned.

With this approach, the correlations $\rho$ were integrated into the data simulation through ${{\varvec{\Sigma}}}_{X}$. The correlation $\rho$ ranged from 0 to 0.95 to represent different levels of correlations between any pair of components (Table

**Table 5.** The Correlation $\rho$ and the Corresponding Correlation Level of FS Endpoint.

$\rho$ in ${\Sigma }_{X}$	Correlation level in ${\Sigma }_{\text{FS}}$	Empirical correlation results (mean ± sd)		
		${\widehat{\rho }}_{{T}_{di}, {T}_{hi}}$	${\widehat{\rho }}_{{T}_{di},{K}_{i}}$	${\widehat{\rho }}_{{T}_{hi},{K}_{i}}$
0	Independent	0.00 ± 0.05	0.00 ± 0.05	0.00 ± 0.05
0.25	Mild	0.22 ± 0.06	0.23 ± 0.05	0.23 ± 0.05
0.5	Moderate	0.45 ± 0.05	0.45 ± 0.04	0.45 ± 0.04
0.75	Moderately high	0.71 ± 0.04	0.68 ± 0.02	0.68 ± 0.03
0.9	High	0.88 ± 0.02	0.81 ± 0.01	0.81 ± 0.01

The empirical results are estimated based on the simulation scenario 1 (under the null) given in Table 2

^5^). Since the data transformations used to generate the two survival times were not linear, the correlation structure, ${{\varvec{\Sigma}}}_{\text{FS}}$, for the FS endpoint (${T}_{di}$, ${T}_{hi}$, ${K}_{i}$) was different from ${{\varvec{\Sigma}}}_{X}$. We evaluated ${{\varvec{\Sigma}}}_{\text{FS}}$ under the following simulation settings (Table 5) and it appeared that the levels of correlations were generally retained for each pair of endpoint components after data transformation.
